# The effect of unpredictability on the perception of breathlessness: a narrative review

**DOI:** 10.3389/fresc.2023.1339072

**Published:** 2024-01-09

**Authors:** Fabien Pavy, Diana M. Torta, Andreas von Leupoldt

**Affiliations:** Research Group Health Psychology, Department of Psychology and Educational Sciences, KU Leuven, Leuven, Belgium

**Keywords:** breathlessness, dyspnea, respiration, unpredictability, uncertainty, fear, anxiety, neural processing

## Abstract

Breathlessness is an aversive bodily sensation impacting millions of people worldwide. It is often highly detrimental for patients and can lead to profound distress and suffering. Notably, unpredictable breathlessness episodes are often reported as being more severe and unpleasant than predictable episodes, but the underlying reasons have not yet been firmly established in experimental studies. This review aimed to summarize the available empirical evidence about the perception of unpredictable breathlessness in the adult population. Specifically, we examined: (1) effects of unpredictable relative to predictable episodes of breathlessness on their perceived intensity and unpleasantness, (2) potentially associated neural and psychophysiological correlates, (3) potentially related factors such as state and trait negative affectivity. Nine studies were identified and integrated in this review, all of them conducted in healthy adult participants. The main finding across studies suggested that unpredictable compared to predictable, breathlessness elicits more frequently states of high fear and distress, which may contribute to amplify the perception of unpredictable breathlessness, especially its unpleasantness. Trait negative affectivity did not seem to directly affect the perception of unpredictable breathlessness. However, it seemed to reinforce state fear and anxiety, hence possible indirect modulatory pathways through these affective states. Studies investigating neural correlates of breathlessness perception and psychophysiological measures did not show clear associations with unpredictability. We discuss the implication of these results for future research and clinical applications, which necessitate further investigations, especially in clinical samples suffering from breathlessness.

## 1 Introduction

Breathlessness, defined as a subjective experience of breathing discomfort which may vary in quality and intensity (1, 2), is a prevalent condition affecting approximately one-fourth of the European adult population (3). Its prevalence increases with aging (3–5), but is not limited to a specific age group as it represents a significant symptom in various respiratory (e.g., asthma, chronic obstructive pulmonary disease) and non-respiratory diseases and disorders (lung cancer, cardiovascular diseases, anxiety, obesity) (5). Its consequences are often highly detrimental for the quality of life of individuals, including difficulties to perform daily tasks, to engage in social activities and to continue their careers and normal lives. Breathlessness is typically experienced as highly aversive and can cause panic and fear of dying which both profoundly undermine well-being (6). Affected individuals are at increased risk of becoming socially isolated (7–9) or to develop affective disorders such as depression and anxiety, which are often associated with the severity of breathlessness (5, 10–14). Moreover, the loss of autonomy of patients and their distress is also demanding for their relatives and caregivers, who frequently develop worries and concerns resulting in reductions in their quality of life (8, 9, 15).

Qualitative studies looking at patient testimonials have proposed to distinguish between continuous and episodic breathlessness (16, 17), the latter referring to a transient exacerbation of the symptom. Episodic breathlessness can manifest predictably, triggered by specific factors known to the patient such as emotions (e.g., panic), physical activities, comorbid diseases (e.g., infections) or environmental circumstances like dust or heat (17–19). It can also occur unpredictably, either because of a lack of discernible triggers or because it emerges at a sudden unusual threshold such as after a faint instead of a strong emotion (16, 18). Unpredictable breathlessness tends to occur less frequently than predictable episodes (18). Yet, unpredictability has been reported by patients to be particularly distressing because (1) it impairs their coping abilities and leads to feelings of loss of control and frustration (9, 18), (2) unpredictable breathlessness is often perceived as more severe and unpleasant (18), and (3) (as a possible consequence of the second point) catastrophizing thoughts and fear of suffocation may trigger panic (6, 18, 19) and initiate a vicious circle as panic contributes to the further aggravation and persistence of breathlessness (14, 16, 20). However, all these observations have been made in clinical contexts and need supporting evidence from controlled experimental contexts to better understand the dynamics and mechanisms influencing the perception of unpredictable breathlessness.

Moreover, an increasing number of studies have investigated the neural mechanisms underlying the perception of breathlessness using different neuroimaging techniques such as functional magnetic resonance imaging (21–24) and electroencephalography (EEG) (25–32). These studies commonly reported activations during experimentally induced breathlessness in affect-related limbic brain areas (22–24) and respective associations with state and trait negative affect (24, 33). Similarly, attentional and affective states were shown to modulate the neural processing of respiratory stimuli (28, 30–32, 34, 35). However, respective interactions with the unpredictability of breathlessness remain poorly understood.

**Figure 1:**
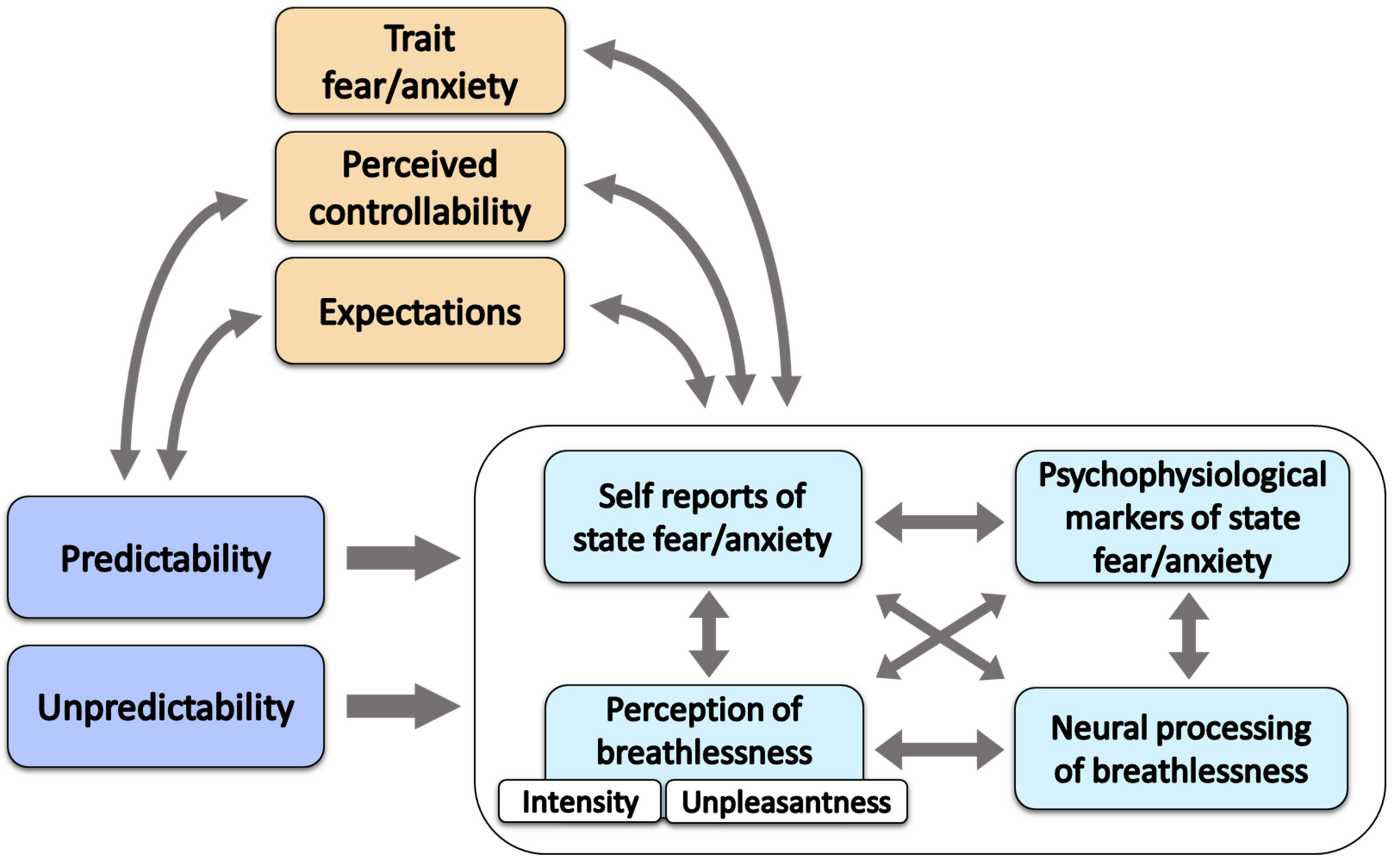
Schematic representation of the reviewed dynamics related to (un)predictability of breathlessness. The arrows in the figure represent possible dynamics. (Un)predictability (darker blue) may influence the components of breathlessness experience, i.e., the perception of breathlessness, the elicited affective states and their psychophysiological and neural markers (light blue). It can also be assumed that these components are interdependent. Trait fear/anxiety, perceived controllability and expectations (orange) can be associated with (un)predictability. For instance, different (un)predictable contexts may elicit different expectations, and less precise expectations can be considered more unpredictable. Moreover, unpredictability and uncontrollability are entangled as knowledge is often required to exert control and to have feelings of control. Trait fear/anxiety, perceived controllability and expectations (orange) can also modulate future breathlessness experiences (light blue) and be modulated by previous breathlessness experiences.

In the present manuscript, we review the currently available experimental evidence about the effect of unpredictability on the perception of breathlessness in the adult population. In addition, we explore potential mechanisms underlying the effect of breathlessness unpredictability such as state and trait fear/anxiety, fear of suffocation and potential psychophysiological and neural correlates (Figure 1). We also briefly discuss other clinically relevant concepts such as controllability and expectations, and make relevant parallels with the literature on unpredictable pain perception. We conclude with a brief overview of the implications for future research and clinical applications.

## 2 Method

We adopted a structured, but not systematic, search strategy that we present hereafter as a good practice statement and to clarify the scope of this narrative review. We searched the PubMed database for relevant articles in English language up to June 2023. The references of the included articles were also screened for other relevant studies. We accepted experimental studies measuring either of the following outcomes: self-reported breathlessness intensity, self-reported breathlessness unpleasantness, and fear-or-anxiety-related psychophysiological and electrophysiological measurements obtained in (un)predictable breathlessness contexts [e.g., electromyographic (EMG) startle response, skin conductance response (SCR), respiratory-related evoked potentials (RREP)]. Studies were valid for inclusion when one condition used more predictable breathlessness stimuli than another condition which was by consequence more unpredictable. Moreover, breathlessness stimuli had to be comparable in terms of type of breathlessness manipulation (e.g., inspiratory resistive loads, CO_2_ enriched-air, complete breathing obstruction…), intensity and duration between the (more) predictable and the (more) unpredictable conditions.

## 3 Results

### 3.1 Identified studies

Nine relevant studies were included in this review (see summary descriptions in Table 1), with a total number of 435 participants (343 females; 92 males, mean age: 21.7 years). The experimental manipulations of breathlessness ranged from inspiratory resistive loads (inspiration difficult but possible) to inspiratory occlusions (inspiration briefly interrupted), to full breathing occlusions (inspiration and exhalation impossible). In some cases, inspiratory resistive loads and inspiratory occlusions were delivered simultaneously whereas in other cases, they were delivered alternately. All studies included self-reports and/or psychophysiological measures (e.g., SCR or EMG startle response) of state fear and anxiety. Five of these studies included measures of trait fear and anxiety (25, 36–38) or trait negative affectivity (26). Lastly, 3 studies included measures of neural activity such as functional magnetic resonance imaging (fMRI) (21) and RREPs (25, 26). Two articles were identified as relevant, but later excluded because of an absence of time interval between the conditioned stimulus CS (CO_2_-enriched air for 5 s) and the unconditioned stimulus US (CO_2_-enriched air for 15 s) in the predictable condition, and because the CS and US were not distinguishable from one another in terms of intensity, thus making the dyspneic CO_2_ stimulation period appear longer in the predictable than in the unpredictable condition (39, 40). Below, we will present the main results of the included studies grouped by outcome domain.

**Table 1:** Basic description of the retrieved studies.

Reference	Subjects and type of design	Predictable condition	Unpredictable condition	Breathlessness stimulus	Main findings
Benke et al. (2018)	*N* = 34 (19 ♀) mean age ± SD = 23 ± 3 years Within subject design	CS (mild IRL, duration: 15 s) immediately followed by the US	Unpaired CS (mild IRL, 15 s) and US	US: maximally tolerable inspiratory resistive load (IRL, duration: 25 s)	High Fear of Suffocation ⤷ Fear Startle CS > ISI (*P* only) ⤷ Fear Startle during ISI: U > P Unpleasantness ratings: U = P
Faull et al. (2016)	*N* = 18 (6 ♀) mean age ± SD = 28 ± 4 years Within subject design	Cue (symbol, duration: 30 s). The IRL appeared 5–15 s after the cue onset, in 100% of the cases. fMRI context	Cue (symbol, duration: 30 s) followed by the IRL in 50% of the cases. fMRI context	IRL (duration 15–25 s)	State NA: P > U Intensity ratings: P > U PAG activated during cues ⤷ High Int. ratings (P only)
Jelinčić et al. (2022)	*N* = 51 (37 ♀) mean age ± SD = 21 ± 3 years Within subject design	Pairs of brief inspiratory occlusions interspersed by 6 IRL. The IRL were always presented one full breath after an auditory cue	Pairs of brief insp. occlusions. interspersed by 6 IRL. 1 or 2 IRL were presented one full breath after an auditory cue. The other IRL were not paired with the cues	• Pairs of brief inspiratory occlusions (occlusion 150 ms, ISI 500 ms) • IRL eliciting very severe unpleasantness	State NA: U > P Int. & unpl. ratings: U = P When neural gating U > P, ⤷ Int & Unpl ratings U > P
Pappens et al. (2012)	*N* = 40 (31 ♀) mean age = 22 years age range: 18–30 years Between subject design	Acquisition phase: CS (non aversive IRL, duration: 8 s) + US + ISI (duration 27–30 s)	Acquisition phase: CS (duration: 8 s) + ISI (duration 27–30 s) + US	US: breathing obstruction applied for 40% of the maximal post-expiratory breath holding time (mean ± SD = 8.8 s ± 1.3 s)	Fear Startle: P = U SCR: P > U in late acquisition ⤷ Expectancy: P > U
Pappens et al. (2013) intero-IFC	*N* = 74 (58 ♀) mean age = 20 years age range: 18–27 years (for EMG startles: *N* = 65, 45 ♀) Between subject design	Acquisition phase: CS (*non aversive IRL*, duration: 8 s) + US + ISI (duration 25–35 s)	Acquisition phase: CS (*non aversive IRL*, duration: 8 s) + ISI (duration 25–35 s) + US	Aversive IRL (3.91 kPa × l/s, duration: 25–35 s)	State NA: U = P Fear Startle: P = U ⤷ P: CS > ISI ⤷ U: CS = ISI
Pappens et al. (2013) extero-IFC	*N* = 42 (34 ♀) mean age = 20 years age range: 18–27 years (for EMG startles: *N* = 65, 45 ♀) Between subject design	Acquisition phase: CS (*neutral picture*, duration: 8 s) + US + ISI (duration 25–35 s)	Acquisition phase: CS (*neutral picture*, duration: 8 s) + ISI (duration 25–35 s) + US	Aversive IRL (3.91 kPa × l/s, duration: 25–35 s)	State NA.: U > P Fear Startle: P = U ⤷ P & U: CS > ISI ⤷ P > U for CS-ISI
Pappens et al. (2015)	*N* = 56 (51 ♀) mean age = 19 years age range: 18–25 years Between subject design	Acquisition phase: CS (non aversive IRL, duration: 8 s) + US + ISI (duration 25–35 s)	Acquisition phase: CS (non aversive IRL, duration: 8 s) + ISI (duration 25–35 s) + US	Aversive IRL (3.91 kPa × l/s/, duration: 30 s)	Fear Startle: P = U SCR: P = U ⤷ Expectancy: P = U
Schroijen et al. (2016)	*N* = 48 (36 ♀) mean age = 26 years age range: 18–58 years Within subject design	Cue (symbol) immediately followed (after cue offset) by breathlessness in 50% of the trials and by nothing in the other 50%	Cue (symbol) followed by breathlessness occurring at any moment in 50% of the trials, and not at all in the other 50%. In practice, when administered breathlessness occurred 8 s after the cue offset	Breathing obstruction applied for 40% of the maximal post-expiratory breath holding time (mean ± SD = 11.32 s ± 2.9 s)	State NA: U > P Fear Startle: P = U High Trait NA ⤷ High Int. ratings ⤷ High Unpl. ratings (P only) High fear of suffocation ⤷ Low Fear Startle (P only) ⤷ High Int. ratings Int. & unpl. ratings: U = P
Tan et al. (2019)	*N* = 32 (22 ♀) mean age ± SD = 25 ± 6 years Within subject design	Brief occlusions every 2–6 breaths (+ forced choice about occlusion duration) interspersed with 10 tone probes immediately followed by a IRL.	Brief occlusions every 2–6 breaths (+ forced choice about occlusion duration) interspersed with 10 tone probes. 10 IRL were unpaired with the tone probes and could occur at any time	• Brief inspiratory occlusions (duration: 160 and 240 ms) • IRL (duration: 2 inspirations)	State NA: U > P when U first High Trait NA: ⤷ High Unpl. ratings ⤷ High State NA (U only) Intensity ratings: U = P Unpleasantness ratings: U > P
von Leupoldt et al. (2021)	*N* = 40 (37 ♀) mean age = 18 years age range: 18–21 years Within subject design	cue (color frame) + IRL (20 s). A pair of brief occlusions and a tone probe were presented during IRL. A 20 s countdown predicted the IRL offset.	cue (color frame) + IRL (13, 20 or 40 s). A pair of brief occlusions and a tone probe were presented during IRL. Random numbers presented instead of countdown. Only 20 s IRL were analyzed.	• Pairs of brief inspiratory occlusions (occlusion 150 ms, ISI 500 ms) • IRL eliciting very severe unpleasantness (for a 20 s duration)	State NA.: U > P High Fear of Suffocation ⤷ State NA: U > P Intensity ratings: U = P Unpleasantness ratings: U > P RREP and neural gating: U = P

CS, conditioned stimulus (cue); IFC, interoceptive fear conditioning; Int, perceived intensity; IRL, inspiratory resistive load; ISI, interstimulus interval; NA, negative affectivity, for trait negative affectivity, a distinction is made between non-specific negative affectivity (labeled trait NA) and fear of suffocation (FoS) which is specifically related to breathlessness; P, predictable condition; RREP, respiratory event-related potential; SCR, skin conductance response; U, unpredictable condition; Unpl., unpleasantness, US, unconditioned stimulus.

### 3.2 State negative affectivity

Across the reviewed studies, results mainly showed that unpredictable breathlessness induces more anxiety and fear than predictable breathlessness (25, 26, 37, 38, 41). For instance, Schroijen et al. (37) used cues to manipulate the predictability and anticipation of breathing occlusions. They found significantly higher anxiety ratings in the condition with unpredictable delayed breathing occlusions compared to the predictable condition with non-delayed breathing occlusions. Specifically, unpredictability and anticipation seems to have reduced the distinction between the threatening phases (during the cue) and the safe phases (interstimulus interval before the cue), which resulted in an increase in fear and anxiety during the safe phases of the unpredictable condition relative to the predictable condition. Similar trends towards higher fear in the condition with unpredictable and delayed inspiratory resistive loads have also been observed by Pappens et al. (41). In the same vein, further manipulations of the (un)predictability of inspiratory resistive loads by von Leupoldt et al. (25) and Jelinčić et al. (26) showed that fear of breathlessness was exacerbated in unpredictable conditions for both onset and duration types of unpredictability. The effect of onset unpredictability can however be mitigated by Tan's et al. results (38) showing that it concerns only the participants who started with the unpredictable condition, and not those who started with the predictable condition. These specific findings may also suggest that experiencing first a predictable context could decrease the threatening aspect of the following unpredictable context, hence reducing unpredictability-elicited fear. Importantly, a study of Faull et al. (21) showed results contrasting with the other studies, with higher anxiety scores in the predictable compared to the unpredictable condition. This may possibly be related to design specificities such as the fact that some elements of unpredictability were also included in the (more) predictable condition. The delivered breathlessness stimuli were induced by inspiratory resistive loads, with a 100% cue contingency in the predictable condition and a 50% cue-contingency in the unpredictable condition. However, both predictable and unpredictable conditions contained variations of anticipation (5–15 s) and stimulus duration (15–25 s) which could have dampened the distinction between predictability and unpredictability. Moreover, this study was conducted in an fMRI scanner, an environment possibly more aversive than usual laboratory settings due to supine position and/or space restriction.

Instead of using (only) subjective reports of fear and anxiety, several studies by Schroijen et al. (37) and Pappens et al. (41–43) included electromyographic measurements of the fear-potentiated startle response [a startle reflex potentiated by fear and anxiety (44)] as an indicator of the successful learning of cue-breathlessness contingencies in fear conditioning designs^1^. None of the breathlessness fear-conditioning studies identified for this review found overall significant differences in the magnitude of the startle responses between the predictable and unpredictable conditions. However, the authors reported that, within the predictable condition, the startle responses were more pronounced during the presentations of the conditioned stimulus^1^ (CS, threatening phase) than during inter-stimulus interval (safe phase). This suggests that the anticipation of an upcoming breathlessness episode during a predictable CS induces a state of fear and vigilance, which disappears during the safe interstimulus intervals (ISI). The self-reports showing an increase in fear during the late as compared to the early phase of the predictable CS presentations further support this idea of an alternance of fear and safety phases in the predictable condition (41). On the contrary, the absence of such a difference in fear between the CS and the interstimulus interval in the unpredictable conditions seems to indicate that fear is maintained during the entire unpredictable condition.

In addition to the fear-potentiated startle responses, Pappens et al. (42) measured skin conductance responses (SCR) as a fear index during the presentation of the CS. They found that the CS elicited higher SCR in the predictable than in the unpredictable condition, especially in the 2nd and 3rd blocks of the acquisition phase (i.e., in the last two blocks). The self-reports of breathlessness expectancy showed that the CS-US contingencies were learned from the 2nd block onward in the predictable condition but not in the unpredictable condition. Therefore, it can be inferred that the more elevated SCR during the predictable CS most likely represented an elevated arousal caused by the learned fear of imminent breathlessness. In a later experiment, Pappens et al. (43), did not observe any significant difference in SCR between the predictable and unpredictable conditions when differences in expectancy were also non-significant.

In short, except for one study (21) the reviewed studies consistently highlight more self-reported fear and anxiety in unpredictable breathlessness contexts. This is especially the case during safe phases (i.e., ISI) rendered less distinguishable from threatening phases by unpredictability. Physiological measures such as SCR have also suggested that the threatening phases (i.e., CS) could sometimes elicit more fear in predictable than unpredictable contexts. This effect seems likely due to fear learning which is facilitated in predictable conditions by the clear cue-breathlessness contingencies.

### 3.3 Trait negative affectivity

A large body of literature has shown associations between trait negative affectivity (especially fear/anxiety and depression) and increased breathlessness severity (5, 10–13, 45–48). Unsurprisingly, similar effects have also been found in the reviewed studies. For instance, Jelinčić et al. (26) measured trait negative affectivity with the Positive And Negative Affect Schedule [PANAS, (49)] and observed that higher scores were associated with higher unpleasantness ratings to the brief inspiratory occlusions. However, they did not find any interaction between trait negative affectivity and unpredictability. Likewise, Schroijen et al. (37) and Tan et al. (38) reported associations between higher anxiety sensitivity and higher breathlessness intensity (37) and breathlessness unpleasantness (38), this independently of the predictability of the stimulus. However, it can be noted that a predictability-related association between anxiety sensitivity and breathlessness unpleasantness was found by Schroijen et al. (37). No such association between anxiety sensitivity and breathlessness unpleasantness was found in the unpredictable condition. Regarding the relationship between trait and state fear/anxiety, Tan et al. (38) observed that higher anxiety sensitivity related to increased ratings of state anxiety in the unpredictable condition, but not in the predictable condition. No association between trait anxiety and state fear/anxiety was found by Schroijen et al. (37).

Fear of suffocation is another affective personality trait to consider when investigating breathlessness perception. Research has demonstrated that fear of suffocation is a more reliable predictor of state fear/anxiety than anxiety sensitivity (50). In the reviewed studies, fear of suffocation has been associated with the amplitude of the fear-potentiated startle response (36), an indicator of state fear and anxiety. In particular, the affective distress in the unpredictable breathlessness condition appeared to be accentuated by high fear of suffocation. Von Leupoldt et al. (25) observed that the participants with high relative to low fear of suffocation showed overall higher state fear and SCR during the breathlessness experiment and showed specifically higher fear reports for unpredictable than predictable breathlessness. In the same vein, Benke et al. (36) found that the participants with high fear of suffocation (compared to those with low fear of suffocation) exhibited higher startle responses during the safe phase (ISI) of the unpredictable compared to the predictable conditions. Additionally, high fear of suffocation has been shown to relate to increased state fear caused by the predictable imminence of breathlessness. In particular, Benke et al. (36) observed in participants with high fear of suffocation higher startle responses during the predictable threatening phase (CS) than during the predictable safe phase (ISI). Together, these results suggest that high fear of suffocation increases state fear and contributes to the maintenance of high levels of fear in unpredictable contexts, whereas in a predictable context, high fear of suffocation transiently increases fear only when breathlessness is imminent. However, it has to be noted that not all research findings are univocal. For example, Schroijen et al. (37) observed a contradictory negative correlation between fear of suffocation and the fear-potentiated startle in the predictable condition. Moreover, they did not find any association between fear of suffocation and self-reported state fear/anxiety. Regarding breathlessness intensity and unpleasantness, von Leupoldt et al. (36) did not find any association with fear of suffocation whereas Schroijen et al. (36) found a significant medium correlation (*r* = 0.38) between fear of suffocation and breathlessness intensity.

In summary, the existing literature on breathlessness unpredictability points at a potential modulatory effect of trait negative affectivity, especially fear of suffocation, on state negative affectivity. Differential modulatory effects in predictable and unpredictable contexts appear often contradictory and require more comprehensive investigations.

### 3.4 Perceived intensity and unpleasantness

Five studies measured the perceived intensity (21, 25, 26, 37, 38) and unpleasantness (25, 26, 36–38) of experimentally-induced predictable and unpredictable breathlessness episodes.

For *breathlessness intensity*, only one study by Faull et al. (21) showed greater ratings for inspiratory resistive loads in the predictable compared to the unpredictable condition. As previously stated, this study denoted from the other studies by the presence of several unpredictability components within the more predictable condition, and by its fMRI scanner environment which may have been somewhat more aversive than usual laboratory settings due to supine position or space restrictions. These potential confounds may possibly explain the heightened perceived breathlessness intensity in the predictable condition in this study, whereas the other studies did not show any significant influences of (un)predictability on breathlessness intensity perception (25, 26, 37, 38).

For *breathlessness unpleasantness*, the studies of Tan et al. (38), and von Leupoldt et al. (25) manipulated respectively the predictability of the onset and offset (= duration) of short episodes of breathlessness induced by inspiratory resistive loads. In both cases, more unpleasantness was found in the unpredictable conditions. Three other experiments measured unpleasantness [valence of the condition in Benke et al. (36); breathlessness unpleasantness in Jelinčić et al. (26) and Schroijen et al. (37)], but they did not find any significant modulation by (un)predictability. Interestingly, the experimental manipulation in the study by Jelinčić et al. (26) may have possibly affected breathlessness ratings. Each participant was administered two types of stimuli: electrocutaneous stimulations and resistive-load-induced breathlessness, in different blocks. These stimuli were initially calibrated to elicit the same level of unpleasantness. However, breathlessness turned out to be rated as less unpleasant (and intense) than the electrocutaneous stimulation during the experimental task. Previous work has shown that a perceptual anchor can create a floor effect in the reported perception of distant items (51). Therefore, it may be questioned whether the electrocutaneous stimulations (possible anchor) hampered unpredictability-driven modulations of breathlessness unpleasantness (distant items).

Overall, enhancing effects of unpredictability on breathlessness perception appear inconsistently and only for the unpleasantness dimension. None of the identified studies report significantly higher perception of breathlessness intensity in unpredictable as compared to predictable conditions. One studies even shows higher breathlessness intensity ratings in the predictable condition (21), but this effect could be caused by potential confounders.

### 3.5 Neural correlates

As compared to self-reports and psychophysiological measurements, recordings of brain activity offer a different perspective on the perception of breathlessness with more emphasis on underlying (cognitive) processes. Only a few studies have investigated the effects of unpredictability on neural correlates of breathlessness perception. One of them, conducted by Faull et al. (21), consisted of an fMRI study focusing on the activation of the periaqueductal grey (PAG) in response to predictable and unpredictable inspiratory resistive loads. The PAG is a nucleus in the midbrain which has been associated with defensive behaviors (52), with fear-anxiety (52, 53) and with pain modulation (52, 53). The PAG has also been shown to be involved in the brain processing of respiration and respiratory sensations (52, 54, 55). Faull et al. (21), showed that some areas of the PAG were activated during predictable breathlessness stimuli (lateral and ventrolateral areas) as well as during their anticipation (ventrolateral areas), but these activations were not significantly different from those in the unpredictable condition. They also report that the activation in the lateral PAG during the anticipation of predictable breathlessness stimuli was correlated with perceived breathlessness intensity, but no such effect could be found for anxiety nor for the activation of the PAG during the inspiratory resistive loads (21). Based on these results, Faull et al. (21) proposed that the PAG could play a role in the modulation of perceived breathlessness, as it does for pain (52, 53). However, they could not find any clear association between activations in the PAG and unpredictability.

The respiratory-related-evoked-potentials (RREP) are another cortical response to breathlessness, measured with an electroencephalogram (EEG). Some evidence showed that increased amplitudes of the RREP are associated with increased breathlessness perception (27, 28, 56). The RREP can also be used to assess the capacity of the brain to filter out redundant and irrelevant information related to respiration, such as the second brief inspiratory occlusion in a pair of occlusions presented within one inspiration (25, 26), a process called neural gating (25, 26, 29, 57). In other words, a redundant breathlessness stimulation would be less deeply processed by the brain, resulting in decreased RREP amplitudes and decreased breathlessness perception (27, 28, 30, 56). In a first study, von Leupoldt et al. (25) used pairs of brief inspiratory occlusions administered during breathing through inspiratory resistive loads of either predictable or unpredictable duration. In spite of having found higher breathlessness unpleasantness in the unpredictable condition, this effect did not translate into higher RREP amplitudes. Moreover, neural gating did not seem to be affected by (un)predictability. However, the methodological choice of administering the pairs of brief occlusions during inspiratory resistive loads may have contributed to shift participants' attention from the occlusions to the loads, resulting in a possible mitigation of subtle effects of unpredictability (25) on RREPs and neural gating. In a second study by Jelinčić et al. (26), the authors avoided this possible incidental attentional capture by administering the paired inspiratory occlusions alternately with (un)predictable inspiratory resistive loads. Although the authors found no main effect of unpredictability on neural gating, they found additional interesting associations between the neural gating and the perception of the brief inspiratory occlusions. Notably, they report that higher neural gating (higher RREP reduction) in the unpredictable condition was associated with higher perceived breathlessness unpleasantness and intensity of the inspiratory occlusions. These results appear counterintuitive since smaller RREPs and higher neural gating are usually associated with reduced breathlessness perception (27, 28, 30, 34, 56). In a previous study with similar counterintuitive findings (58), the authors hypothesized that, in other sensory modalities, reduced amplitudes of event-related potentials for redundant stimulations, as observed with neural gating, relate more to a decrease in saliency than to a reduction of perceived intensity (59–61). Therefore, it may be argued that the neural gating of respiratory sensations is also impacted by saliency, and perhaps only influenced by breathlessness perception in specific circumstances which remain to be identified.

Overall, the few studies exploring the neural correlates of breathlessness perception, did not find clear cut results regarding the unpredictability of breathlessness. It appears that some measures of brain activity, such as the amplitudes of event-related potentials (RREP) and the activation of the PAG, may be related to breathlessness perception, as is the case for another aversive sensation: pain. However, results for breathlessness unpredictability are scarce and sometimes surprisingly contradictory, thus pointing at the complex interplay between different cognitive processes (e.g., attention, saliency, perception) that need to be further disentangled to better understand neural correlates of breathlessness unpredictability.

## 4 Discussion

### 4.1 Summary of main findings

The most consistent finding across studies was that states of increased fear/anxiety were more frequently observed in unpredictable breathlessness conditions. The psychophysiological markers suggested that fear/anxiety is sustained during entire unpredictable conditions, but that it alternates between lower and higher states in predictable conditions depending on whether the participants experience a safe phase or a cue predicting imminent breathlessness. More constant fear/anxiety without clear safe phases may possibly explain why unpredictable breathlessness is often perceived as more unpleasant or intense in clinical (18) than non-clinical samples (25, 38).

Trait anxiety has often been associated with increased state fear/anxiety as well as with overall increased breathlessness perception. However, it does not seem to directly influence in a differential manner predictable and unpredictable episodes of breathlessness. Similarly, the results regarding fear of suffocation are not clear-cut, but suggest that it can increase state fear in both the predictable and unpredictable conditions. In other words, indirect modulatory pathways of trait negative affectivity via short-lasting affective states may exist, but require further investigations.

The reviewed experimental studies suggest that unpredictability impacts more the affective than the sensory dimension of breathlessness. Specifically, higher ratings in the unpredictable than predictable condition were more frequently reported for breathlessness unpleasantness than breathlessness intensity. However, several studies were not able to show such findings, in some cases perhaps because of differences in the employed designs and stimuli, requiring further research efforts. Moreover, observed effects of breathlessness unpredictability have not yet been clearly found to relate to neural processing patterns in specific brain areas nor to the neural gating of respiratory sensations. Neural gating may relate mainly to saliency processes (58), with effects on breathlessness perception only in some specific cases, but these hypotheses remain to be empirically confirmed.

### 4.2 Implication for future research and clinical practice

Given the sometimes contrasting or null findings observed in the reviewed studies, future research on the effects of unpredictability on the perception of breathlessness is needed. For example, future studies should further examine potential brain mechanisms involved with unpredictability and clarify the potential moderating role of fear of suffocation. Moreover, direct comparisons between different qualities of breathlessness stimuli (e.g., exercise-induced, CO_2_-induced) may potentially reveal different effects of unpredictability. Importantly, the reviewed studies included exclusively healthy volunteers whose perception of breathlessness may not fully reflect the experience of individuals afflicted with breathlessness. Thus, studies in different clinical samples suffering from breathlessness are required, especially studies in clinical settings as they would be informative to explore effects of (un)predictability during treatments of breathlessness, for example during exercise in rehabilitation contexts.

For pain, another aversive bodily sensation, evidence has shown that unpredictability does not always directly enhance pain perception (62–64). Instead, several variables including pain expectations can partially explain the relationship between unpredictability and pain perception (62–64). Such effects have not yet been systematically confirmed for breathlessness, although research has already repeatedly suggested comparable influences of expectations on breathlessness perception in both clinical and non-clinical samples (22, 65–70). Negative expectations are also an important element of catastrophizing, itself associated with worse quality of life (71). This highlights the need for future investigations into the effects of expectations on breathlessness.

In the reviewed studies, responses to unpredictable breathlessness did not seem to differ with respect to the type of unpredictability (onset, offset). However, effects of unpredictable intensities of breathlessness have not yet been investigated. According to research with other aversive bodily sensations such as pain (63, 64), this type of manipulation of unpredictability is more likely to create differences in pain perception between the predictable and unpredictable conditions because of underlying differences in expected pain intensities. In the absence of clear differences in expectations, a recent meta-analysis revealed that unpredictability did not significantly influence pain perception, and that for all types of unpredictability (onset, duration, intensity, location) (72). Whether similar effects would hold for breathlessness is currently unknown, warranting future studies. The aforementioned meta-analyses (72) also presented significant moderating effects of state negative affectivity on unpredictable pain perception echoing the present findings for breathlessness.

Controllability is entangled with predictability (73). Not all predictable events would be controllable, but controllability would require some minimal knowledge about the event (e.g., onset, offset, intensity…), therefore some predictability (73). The same reasoning implies that a completely unpredictable event is also uncontrollable. This question about the contingency between uncontrollability and unpredictability is of major importance since higher perceived control over the course of a respiratory disease has been regularly associated with lower symptom severity, lower depression and anxiety and a better quality of life (74–78). The beliefs about the lack of control on breathlessness may also cause panic (19, 79), a highly aversive state which can further increase or maintain breathlessness. Another implication could be that the detrimental effects attributed to unpredictability instead originate from uncontrollability. Therefore, careful considerations about potentially confounding effects of uncontrollability should be an integral part of future research on unpredictability, also in the field of breathlessness.

Research on unpredictability is particularly relevant to reduce the burden associated with breathlessness and to improve the quality of life of patients, two key objectives of pulmonary rehabilitation. Reducing the perceived unpredictability of breathlessness may also potentially decrease negative affect and, through the latter, breathlessness experiences (5, 6, 14, 16, 18–20). Moreover, this may encourage individuals to be more physically active and to re-engage into social activities (7–9, 18). The present review notably suggests that the absence of clear safe phases in unpredictable contexts is likely responsible for the maintenance of heightened negative affective states, hence a possible treatment target. Controllability, because of its entanglement with unpredictability, offers another possible treatment target. Restoring perceived control over breathlessness [e.g., control over catastrophizing thoughts, emotions, coping strategies… (19, 80),], as already assessed in some pulmonary rehabilitation programs (81), may possibly reduce perceived unpredictability. Yet, it has to be noted that controllability might not be beneficial to all individuals, with sometimes positive effects only for male (82) or less fearful patients (83). All these findings emphasize the need for more research on treatments, including unpredictability management.

## 5 Conclusion

Overall, available experimental studies about breathlessness and unpredictability are still rare and results are often not uniform. The current review provides some preliminary answers to understand why unpredictable breathlessness episodes may be particularly distressing for patients. A constant fear and unrest in unpredictable conditions seems associated with the subjective exacerbation of breathlessness, especially its unpleasantness. The known effects of trait negative affectivity on breathlessness do not appear to depend on (un)predictability. However, they suggest possible indirect effects on the perception of unpredictable breathlessness through reinforcements of state fear and anxiety. Taken together, the present observations on the unpredictability of breathlessness remain to be confirmed and extended by further investigations on different types of unpredictability and on its associations with expectations and uncontrollability, especially in clinical samples and treatment contexts. These investigations should also include studies on neural and psychophysiological correlates of unpredictable breathlessness.

## Footnotes

^1^In conditioning studies, a neutral cue (conditioned stimulus CS) is repeatedly presented with an aversive stimulus [unconditioned stimulus US, here inspiratory resistive loads (46, 48) or breathing obstructions (23, 47)] causing fear and anxiety. After an acquisition phase, the neutral cue becomes aversive and able to elicit fear and anxiety, even when the breathlessness stimulus is not jointly presented.
