# Erratum: The effect of unpredictability on the perception of breathlessness: a narrative review

**DOI:** 10.3389/fresc.2024.1427391

**Published:** 2024-05-14

**Authors:** Frontiers Production Office

**Affiliations:** Frontiers Media SA, Lausanne, Switzerland

**Keywords:** breathlessness, dyspnea, respiration, unpredictability, uncertainty, fear, anxiety, neural processing

An erratum on The effect of unpredictability on the perception of breathlessness: a narrative review By Pavy F, Torta DM and von Leupoldt A (2024). Front. Rehabil. Sci. 4:1339072. doi: 10.3389/fresc.2023.1339072

Due to a production error, the corresponding author was incorrectly listed as Andreas von Leupoldt instead of Fabien Pavy.

The publisher apologizes for this mistake.

The original version of this article has been updated.

